# MERGE: A model for multi-input biomedical federated learning

**DOI:** 10.1016/j.patter.2023.100856

**Published:** 2023-10-06

**Authors:** Bruno Casella, Walter Riviera, Marco Aldinucci, Gloria Menegaz

**Affiliations:** 1 Department of Computer Science, University of Verona, 37134 Verona, Italy; 2 Department of Computer Science, University of Turin, 10149 Turin, Italy; 3 Department of Engineering for Innovation Medicine, University of Verona, 37134 Verona, Italy

**Keywords:** multi-input classification, federated learning, mixed-data deep learning, federated classification, biomedical imaging

## Abstract

Driven by the deep learning (DL) revolution, artificial intelligence (AI) has become a fundamental tool for many biomedical tasks, including analyzing and classifying diagnostic images. Imaging, however, is not the only source of information. Tabular data, such as personal and genomic data and blood test results, are routinely collected but rarely considered in DL pipelines. Nevertheless, DL requires large datasets that often must be pooled from different institutions, raising non-trivial privacy concerns. Federated learning (FL) is a cooperative learning paradigm that aims to address these issues by moving models instead of data across different institutions. Here, we present a federated multi-input architecture using images and tabular data as a methodology to enhance model performance while preserving data privacy. We evaluated it on two showcases: the prognosis of COVID-19 and patients’ stratification in Alzheimer’s disease, providing evidence of enhanced accuracy and F1 scores against single-input models and improved generalizability against non-federated models.

## Highlights

• This study demonstrates the advantages of a multi-input federated architecture
• The proposed architecture leverages images and tabular data to improve classification
• Improvements in classification include accuracy and F1 score
• The approach has been tested on 2D (CoViD-CXR) and 3D (ADNI) datasets

## The bigger picture

Deep learning models must be trained with large datasets, which often requires pooling data from different sites and sources. In research fields dealing with sensitive information subject to data regulations, such as biomedical research, data pooling can generate concerns about data access and sharing across institutions, which can affect performance, energy consumption, privacy, and security. Federated learning is a cooperative learning paradigm that addresses such concerns by sharing models instead of data across different institutions.

## Teaser

This study proposes a multi-input federated learning (FL) approach to improve the performance of deep learning (DL) classification tasks while maintaining the inherent advantages of FL architectures. The authors first demonstrate how a DL model can handle input data of different nature simultaneously, such as images (2D and 3D) and tabular records. They then show how the federated multi-input model improves the performance and generalizability of non-federated models while preserving the security and data protection properties peculiar to FL.

## Introduction

Artificial intelligence techniques, such as machine learning (ML) and deep learning (DL), are increasingly exploited as tools to address challenges in various research fields, including the biomedical one. One of the strengths of ML models is their capability to capture hidden and complex relationships in multi-dimensional data. They have been explored for several tasks, including disease classification,^1^^,^^2^^,^^3^ human body segmentation,^4^^,^^5^^,^^6^^,^^7^ the definition of diagnostic scores,^8^ drug discovery,^9^^,^^10^ and data augmentation through the generation of synthetic samples.^11^^,^^12^^,^^13^^,^^14^ While most of these examples concern medical images, such as magnetic resonance imaging (MRI), X-rays, or other types of body scans, images are not the only type of data available in hospitals and clinical laboratories, which routinely collect various diagnostic data: time series coming from electrocardiograms or devices for monitoring vital signs, video from cameras recording patients’ movements and positions overnight or during a rehabilitation therapy, and text or tabular data coming from surveys and administrative and clinical records. This constitutes a rich and heterogeneous data source that is used by clinicians to elaborate on diagnosis and prognosis.

Although these data are different, they have one common feature: they are critical data for privacy and security and must be treated appropriately. Examples of data regulations explicitly created for managing the access and use of health data are the Health Insurance Portability and Accountability Act (HIPAA)^15^ in the USA and the Protection of Personal Information Act (POPIA).^16^ While protecting sensitive information is a critical mission from a governance perspective, from an artificial intelligence (AI) perspective, introducing all these regulations limits data access.

Computing paradigms like federated learning (FL)^17^ can help address this challenge.^18^ In an FL scenario, multiple institutions $C_{i}$ holding proprietary and critical data collaborate to train a global AI model *M*. The crucial aspect of the FL is that data belonging to a specific $C_{i}$ never exit from the IT facility of its owner. Instead of sharing or exchanging data, different institutions iteratively aggregate local models in a single global model *M* that approximates the one that could be achieved by gathering all data in a single data lake.

FL is generally categorized along two main approaches: horizontal (HFL) and vertical (VFL) FL.^19^ In both cases, multiple institutions $C_{i}$ holding proprietary data are willing to train a shared model while never sharing their sensitive information.

**Figure 1:**
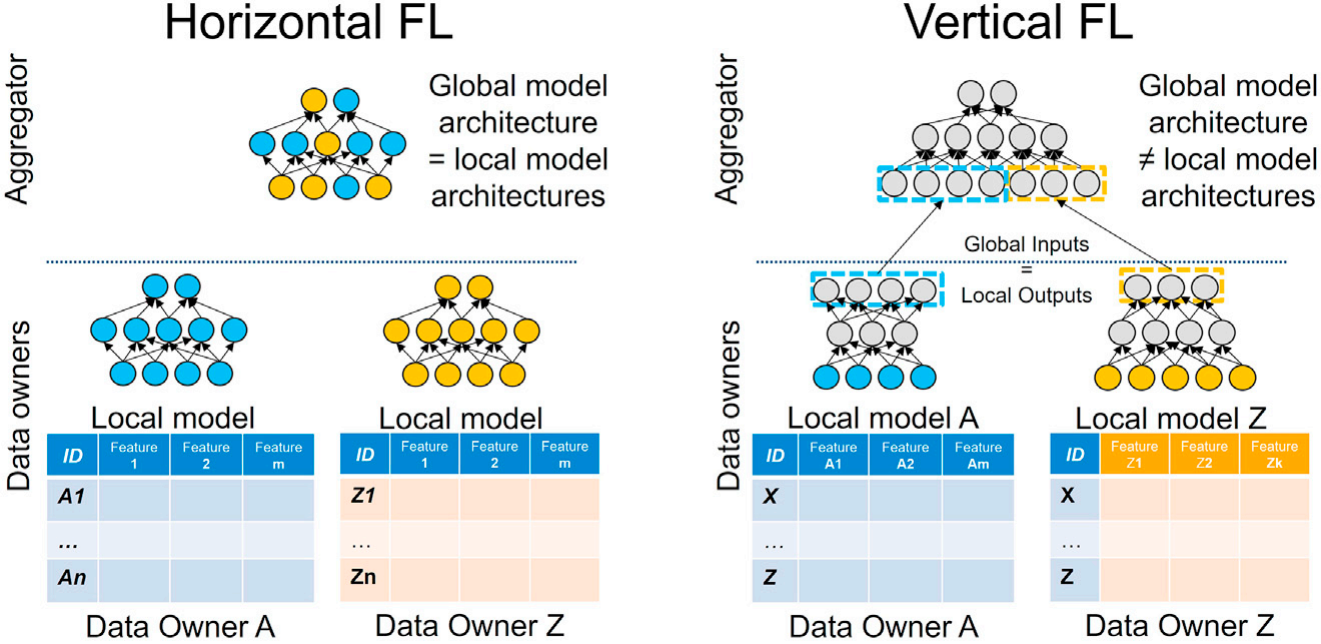
FL settings (A) HFL, with collaborating institutions sharing the same features space but different samples. In this setting, only one model is being trained locally by each institution that is also shared and aggregated. (B) VFL, with collaborating institutions having different features for the same samples. In this case, each $C_{i}$ has its model that flows into a unified 2nd-level model shared across the federation.

The main difference between HFL and VFL resides in the assumption of how data are split among $C_{i}$. In the HFL, the basic assumption is that each $C_{i}$ taking part in the federation has the same feature space (i.e., data format, like MRIs) but different sample instances (i.e., different patients). Conversely, each $C_{i}$ contributes to the federation in the VFL scenario leveraging different data with different feature spaces while the sample instances are the same. An example of HFL and VFL settings can be found in Figure 1.

In this work, we propose a multi-input model that can handle different types of data, namely images and tabular text, trained by following the orchestration paradigm of the HFL schema. Multi-input FL tasks are usually implemented as VFL by design. In this case, multiple models are specifically tailored to the different data sources as shown in Figure 1.

In this context, the proposed solution solves the trade-off between using diverse data to train input-specific models (VFL) and gathering homogeneous data from different populations (HFL), better reflecting the needs encountered in multi-centric studies.

In addition, we demonstrate that our approach is helpful for medical image classification tasks and provide evidence of how our federated multi-input model can outperform the typical single-input models. For this, we focused on two case studies addressing core issues in the field, i.e., COVID-19 prognosis and classification of patients with Alzheimer’s disease, relying on the COVID-19 chest X-ray data (CXR)^20^ (2D data) and the Alzheimer’s Disease Neuroimaging Initiative (ADNI) studies^21^ (3D data), respectively.

The scientific contributions of this work are the following.

• We propose an approach to translate FL multi-input classification tasks from a complex VFL to a more straightforward HFL orchestration.
• We demonstrate how the proposed approach can effectively capture knowledge from multiple data sources, even when exploiting standard (i.e., not custom) models.

In summary, this work proposes an example of a federated multi-input model for classification orchestrated following an HFL schema. The proposed framework is open and flexible: it could be used with different neural network architectures and training algorithms.

Notice that outperforming the state of the art in the classification tasks was not within the scope of this contribution. We are aware that choosing the best model for the problem at hand would lead to better performance in terms of the classification scores. We leave this issue for further investigation.

In line with open-science initiatives, to help reproduce our results and facilitate the customization of the model, we provide the code and instructions on how to use it in the experimental procedures.

### FL in the biomedical field

In recent years, the evolution of ML methods has been dominated by the need to improve the performance of models up to levels that can be used for practical purposes. The introduction of DL has been a real revolution for many tasks, especially for image and text analysis. Unfortunately, DL is data hungry: large-size DL models require more and more data to be trained, and pooling data are often needed to build an appropriate dataset. Pooling increases the model generalizability,^22^^,^^23^ i.e., the ability of a model to deal with unseen data reliably.^24^ Model generalizability has been extensively studied in ML literature.^25^^,^^26^^,^^27^^,^^28^^,^^29^ The studies show how higher exposure to data can increase generalizability, hence the need for real-life federations.^25^^,^^26^^,^^27^^,^^28^^,^^29^ FL provides a viable solution by enabling the virtual pooling of different datasets while maintaining data privacy and allowing the model to learn features appearing in examples from different data owners.

An example of an HFL setting could be represented by a set of institutions, like hospitals, aiming to train a shared model (i.e., for brain tumor segmentation) by leveraging MRI scans. In this scenario, the assumption is that each $C_{i}$ would be able to provide MRI scans of their patients. However, in a real-world scenario, patients differ from hospital to hospital, leading to a non-i.i.d. data distribution known for degrading FL performance.^30^

Despite being relatively new, FL has already demonstrated its value in addressing the generalizability challenge.^31^^,^^32^ Due to the high sensitivity of the data, many other works have been performed to explore FL in the biomedical field.^23^^,^^33^^,^^34^ While the effectiveness of FL in increasing the model generalizability has already been proven, finding the best way to aggregate the contributions from all the data owners remains an open challenge.

### Multi-input classification

Using multiple input sources to perform a classification task is a well-known idea in literature.^35^ The method is referred to with different names like multi-modal,^36^ multi-view,^37^ multi-channel,^38^ or mixed-data ML.^39^ For the sake of clarity, we are going to refer to this topic using the multi-input label. One of the driving factors beyond our adoption of multi-input AI approaches is data availability.

Several multi-modal datasets can be found publicly.^40^^,^^41^^,^^42^^,^^43^ In the biomedical field, coupling different types of images is a common practice. Classification of breast tumor^44^ and Alzheimer’s disease (AD)^45^ and the fusion of learning strategies based on different views of the same image^46^ are some examples.

In other cases, the fusion involves different data types, like time series,^47^ or features extracted from different sources.^48^^,^^49^ Multi-input models have also been tested in contexts other than the biomedical field. For example, a recent work^50^ proposes MI-DCNN, a complete end-to-end multi-input convolutional neural network that can take full advantage of multi-modal physiological signals and automatically complete the process from feature extraction to emotion classification simultaneously.

Regardless of the data type being considered, according to Sleeman et al.,^36^ co-training and co-regularization are the two possible settings of a multi-input-based approach. The co-training refers to those problems in which the information from one source can help to estimate missing details (like labels) on the other, as happens in semi-supervised learning.^51^^,^^52^ Concerning FL, the co-training setting can be easily mapped into the VFL scenario. In the co-regularization case, the various input sources are considered contributors for deriving a common descriptor for representing the instances of the problem being addressed. In this last case, the fusion occurs within the classification model itself after each input has been encoded independently.^53^ For this work, we relied on the co-training setting to train models in an HFL architecture.

### Federated multi-input

The use of multi-input models in an FL setting to enhance generalizability is still narrowly explored in literature. Most related works propose the multi-input approach to support a VFL setting. Huang et al.^54^ describe a scenario where *M* views are distributed across *M* devices. The same considerations apply to other recent works.^55^^,^^56^^,^^57^

In the biomedical environment, Che et al.^58^ propose an approach that can work in both HFL and VFL settings. However, the main focus is pipeline orchestration and data leakage prevention, not generalization. Furthermore, the examples provided refer to preserving the privacy of sequential data, like real-world keyboard data, collected from the BiAffect study.^59^ Qayyum et al.^57^ propose an FL approach where the clinical institutions are organized into two clusters depending on what type of data they own: X-ray data cluster and ultrasound data cluster. Mahbub Ul and Rahim^60^ present an FL multi-input approach working on the Internet of Medical Things (IoMT). However, as claimed by the authors, the main focus is to study the heterogeneity of the hardware equipment used to simulate the different clients (medical institutions) for performing evaluations on time-series models. Regarding the tasks, a recent work^61^ focuses on classifying signals from Internet-of-Things (IoT) devices using autoencoders to extract common representations from the different data sources. However, this contribution is not directly linked to the biomedical field. Bernecker et al.^62^ tackle the liver segmentation problem by proposing a multi-input normalization technique, which tries to encode computed tomography (CT) and MRI scans into a common representation.

This work proposes a multi-input model to enhance classification tasks in an HFL setting. The ultimate ambition is not to provide a specific model architecture but to introduce a methodology that combines multiple input sources to improve classification performance for an HFL pipeline. The model itself is meant to be a proof of concept that is open and flexible such that it can be easily customized for other tasks in different domains. We demonstrate the feasibility of our approach by evaluating the model on two classification tasks: prognosis of COVID-19^20^ disease (2D data) and patient classification in AD, relying on the ADNI initiative^21^ (3D data). While our final goal is not to improve the state-of-the-art performance for the two specific problems, it is essential to highlight how the two considered challenges are open and might benefit from the proposed approach.^63^ Alternative routes to solve the COVID-19 classification task have been explored by considering different feature extraction techniques.^64^^,^^65^ Similar considerations apply to the classification of AD. Many works demonstrate multiple efforts in addressing the challenge by using transfer-learning,^66^ multi-modal,^67^ and multi-input techniques.^68^^,^^69^^,^^70^ However, these are still hot topics, and exploiting FL to preserve data privacy while keeping competitive performance would mark a step forward in the field.

In summary, the few works available in the literature that implement multi-input models in federated contexts either refer directly to the VFL setting, are unrelated to the biomedical environment, or do not address the classification task. This article addresses the problem of having a multi-input classification model in an HFL setting in the biomedical field.

## Results

In this work, we propose leveraging different data types, i.e., images and tabular data, to improve testing accuracy and the F1 score of federated models. Results show how the federated version of the multi-input model can outperform baseline single-input models and increase overall generalizability compared to equivalent centralized (non-federated) settings.

We experimentally evaluated our approach on two different datasets: the CoViD-CXR dataset^20^ (2D) and the ADNI dataset^21^ (3D). The first is publicly available, while the second can be accessed under soft licensing conditions.

For both case studies, we ran several experiments involving training multiple models in multiple settings. More precisely, we evaluated the performance by comparing the classification results obtained by three model architectures, depending on three types of input.

• Image-only input, using a ResNet-18^71^ model.
• Tabular-only input, leveraging a multi-layer perceptron (MLP)^72^ model.
• Multi-input, using a concatenation layer to fuse the outputs of the previous two models (image only and tabular only) as shown in Figure 3.

In the image-only and multi-input cases, we used the 2D version of ResNet-18 for the experiments based on the CoViD-19 CXR dataset^20^ and the 3D version for experiments on the ADNI dataset.^21^

Experiments were conducted to analyze the DL model behavior in the following settings.

**Figure 2:**
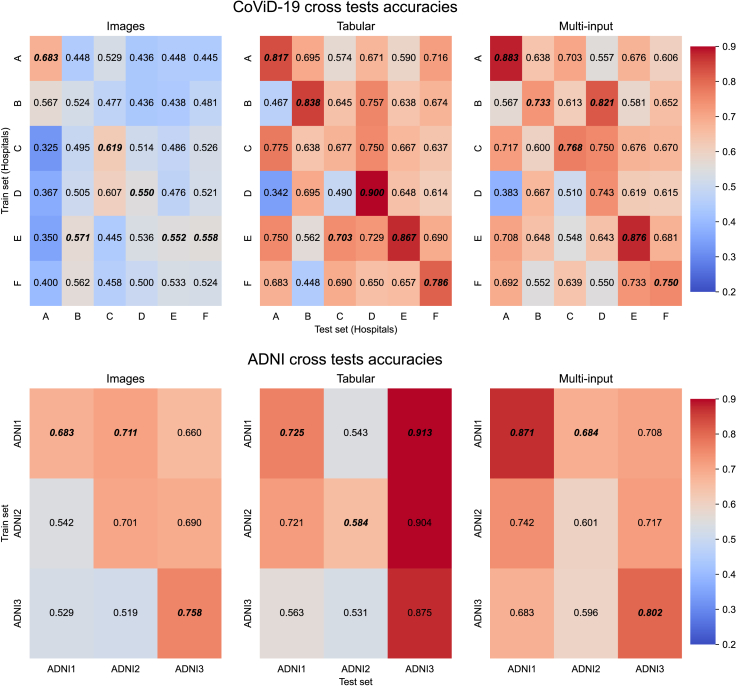
Accuracy values obtained by testing the isolated models on data belonging to other institutions
• Centralized: to simulate collaboration among institutions without privacy constraints. Models were trained on a unified dataset by forcing all data to be hosted in the same computing facility. Results are shown in Table 3.

• Isolated: to simulate no collaboration among institutions. Models were trained independently and tested using data belonging to every single organization. In this scenario, we have three models (one for each input type) for each of the six hospitals of the COVID-19 dataset^20^ and each ADNI (ADNI1, ADNI2, and ADNI3)^21^ study. Results are shown in Tables 1 and 2. Moreover, isolated models have also been tested on the data of other organizations in order to discuss generalizability properties. Results are shown in Figure 2.

**Table 1:** Accuracy in the isolated setting with COVID dataset

Input	COVID-19 hospital					
	A	B	C	D	E	F
Only images	0.683±0.06	0.524±0.06	0.619±0.09	0.550±0.13	0.552±0.06	0.524±0.04
Only tabular	0.817±0.03	0.838±0.04∗	0.677±0.05	0.900±0.03∗	0.867±0.06	0.786±0.02∗
Multi-input	0.883±0.03∗	0.733±0.04	0.768±0.06∗	0.743±0.09	0.876±0.07∗	0.750±0.02

Results (mean ± standard deviation) obtained with 5-fold cross-validation (centralized) and five averaged runs (federated). For each experiment setting (column), we indicated the best-performing model with an asterisk.

**Table 2:** Accuracy in the isolated setting with ADNI dataset

Input	ADNI1	ADNI2	ADNI3
Only images	0.521±0.02	0.717±0.06∗	0.756±0.02
Only tabular	0.725±0.00	0.615±0.03	0.881±0.03∗
Multi-input	0.871±0.07∗	0.605±0.05	0.808±0.08

**Table 3:** Accuracy in centralized and federated setting

Input	COVID-19 CXR		ADNI	
	Centralized	Federated	Centralized	Federated
Only images	0.731±0.06	0.558±0.02	0.777±0.01	0.855±0.04
Only tabular	0.740±0.03∗	0.696±0.02	0.638±0.02	0.715±0.03
Multi-input	0.733±0.01	0.734±0.01∗	0.811±0.03∗	0.866±0.02∗

Results (mean ± standard deviation) obtained with 5-fold cross-validation (centralized) and five averaged runs (federated). For each experiment setting (column), we indicated the best-performing model with an asterisk.
• Federated: to simulate the collaboration among institutions subject to privacy constraints. Models were trained following an HFL orchestration schema. Results are shown in Table 3.
• Isolated and centralized experiments: the datasets were split into three subsets for training, validation, and testing using 80%, 10%, and 10% quotas, respectively. We fixed the test set and used the train and validation sets to train the models on five stratified folds in each centralized experiment. The reported accuracy values were obtained by evaluating the best model with the best validation accuracy on the test set.
• Federated experiments: the datasets were partitioned only in training and testing sets for two reasons. Firstly, since each $C_{i}$ would perform only one iteration before sending the model back to the aggregator, running a cross-validation step would not bring any benefits, as there would not be any iterative process to optimize. Then, the tool used for running the federated experiments would not allow a 5-fold cross-validation phase without massive intervention to the low-level code. To have comparable results, we preserved the percentage of training data by splitting the dataset into train (80% of the entire dataset) and test (the remaining 20%) sets, respectively. Further considerations are shared in the discussion subsection. We ran the federated experiments five times to ensure that the performance would not depend on a specific data split, and we averaged the results.

## Discussion

This article proposes leveraging multiple inputs, i.e., images and tabular datasets, to improve testing accuracy and the F1 score of models trained with HFL architecture. To assess the quality of this approach, we structured the investigation in three steps: first, we assessed the generalizability of each model in the isolated setting; second, we evaluated how the performance would improve when pooling the data together in a centralized setting, and third, we repeated the evaluation in a federated setting.

For the first step, we evaluated each model trained on the data of a specific COVID-19 hospital (or ADNI partition) on a testing set from each of the other COVID-19 hospitals (or ADNI partitions). Results are reported in Figure 2. The diagonals show the accuracy obtained when testing a model with data exhibiting the same training set distribution. Figure 2 shows how isolated models do not generalize well on new data. Indeed, when tested on data from other institutions, the accuracy drops significantly compared with when tested on local proprietary data. One exception is represented by the ADNI evaluation on tabular data, where the highest performance is obtained when testing the model using ADNI3. This behavior is due to the high-class imbalance of ADNI3, as reported in Table 4.

**Table 4:** Main demographic and clinical data for the three ADNI study cohorts

ADNI	Samples	AD (%)	CN (%)	Age (avg ± SD)	Gender		APOE4		
					F	M	Type 0	Type 1	Type 2
1	411	184 (44.77)	227 (55.23)	75.58 ± 6.21	198	213	229	143	39
2	288	143 (49.65)	145 (50.35)	73.69 ± 7.35	130	158	149	107	32
3	262	51 (19.47)	211 (80.53)	72.01 ± 6.44	136	126	169	75	18

Age is reported as mean ± standard deviation values and gender as the number of males/females, while APOE4 refers to the number of ε4 alleles (0, 1, or 2, respectively). avg, average; SD, standard deviation.

For the second step, we evaluated models in a centralized setting, gathering all samples in a single data lake to simulate collaboration among institutions without privacy constraints. As expected, pooling data from several institutions can improve the generalizability of the models. Table 3 shows how the centralized models generally perform better than the isolated models (Figure 2). This aspect can be appreciated by comparing the centralized accuracy with all the off-diagonal values of the two tables. As previously noted, ADNI3 class imbalance represents an exception to this consideration, as confirmed by looking at the F1 score obtained by the three models trained on ADNI3 in Tables 5 and 6. Overall, improvements in model generalizability are confirmed by looking at the F1-score values in the isolated and centralized settings, reported in Tables 5, 6 and 7, respectively. Similarly to the accuracy values, the same conclusions can be drawn for the F1 scores.

**Table 5:** F1 score in the isolated setting with COVID data

Input	COVID-19 hospital					
	A	B	C	D	E	F
Only images	0.808±0.03	0.364±0.31	0.616±0.13	0.147±0.23	0.314±0.32	0.260±0.33
Only tabular	0.893±0.01	0.800±0.40∗	0.686±0.08	0.943±0.08∗	0.848±0.11	0.793±0.05∗
Multi-input	0.978±0.04∗	0.200±0.40	0.826±0.11∗	0.600±0.49	1.00±0.00∗	0.687±0.11

Results (mean ± standard deviation) obtained with 5-fold cross-validation (centralized) and five averaged runs (federated). For each experiment setting (column), we indicated the best-performing model with an asterisk.

**Table 6:** F1 score in the isolated setting with ADNI data

Input	ADNI1	ADNI2	ADNI3
Only images	0.289±0.29	0.667±0.08∗	0.081±0.13
Only tabular	0.684±0.02	0.542±0.05	0.173±0.12∗
Multi-input	0.703±0.03∗	0.463±0.13	0.058±0.07

**Table 7:** F1 score in centralized and federated setting

Input	CoViD-19 CXR		ADNI	
	Centralized	Federated	Centralized	Federated
Only images	0.515±0.30	0.197±0.14	0.662±0.03	0.735±0.05
Only tabular	0.562±0.28∗	0.623±0.01	0.379±0.02	0.573±0.04
Multi-input	0.520±0.30	0.636±0.05∗	0.742±0.03∗	0.745±0.04∗

Results (mean ± standard deviation) obtained with 5-fold cross-validation (centralized) and five averaged runs (federated). For each experiment setting (column), we indicated the best-performing model with an asterisk.

For the third step, we compared the federated and the centralized settings. Notice that the centralized setting is an upper bound for the federated setting in terms of model performance since it can simulate the federated one (even if this is not usually how it is used). However, in many real-life scenarios, the centralized setting is unrealistic due to data regulations preventing different institutions from sharing data. For this reason, it is fundamental that FL performance remains aligned with centralized performance. Sheller et al.^34^ demonstrated that FL approaches can enhance the model’s generalizability by leveraging multiple datasets, but they suffer from slower convergence and slightly inferior performance than centralized solutions. For the COVID-19 dataset, the single-input results presented in Table 3 confirm this behavior. However, our proposed FL approach using multi-input models outperforms the federated single-input versions and remains aligned with the centralized counterpart. For the ADNI dataset, all the federated models outperform the centralized counterparts. While counterintuitive, this can be explained by looking at the different data partitions between the two experimental settings. Indeed, the centralized values are obtained by querying the best validation model on the test set, using an 80%/10%/10% train/validation/test split instead of the 80%/20% train/test split used for the federated setting without cross-validation. Validation sets are usually helpful in identifying and selecting the best-performing model among various epochs of the training process. For our federated experiments, since each collaborator $C_{i}$ would perform only one training epoch before sending the model back to the aggregator, running a cross-validation step would not bring any benefits, as there would not be any iterative process to optimize or best model to select. Nonetheless, running multiple-fold cross-validation in a federated setting would have required a massive refactoring of the selected tool, as this feature is unavailable. To ease the results’ reproducibility, we fixed the number of epochs per aggregation round to one epoch. As described in Casella et al.,^73^ fine-tuning epochs per round might slightly improve the convergence speed and the model’s performance.

In real-life scenarios, hospitals willing to train an FL model would instead use common (ideally public) external sources to test their model instead of sharing a subset of their proprietary datasets. By choosing the 80%/20% training/test split, we could simulate a more realistic scenario by keeping the testing set external to the training process.

### Conclusions

This article proposes a new approach for addressing classification tasks for the multi-centric studies through privacy-preserving ML models. The method introduces an HFL setting with the advantage of leveraging multiple input sources. The basic assumption for the proposed approach is that each $C_{i}$ taking part in the federation has both data types, images and tabular data, locally available and accessible. In particular, we tested our method on two classification tasks: prognosis of COVID-19 disease from CXR (CoViD-CXR dataset^20^) and detection of AD from neuroimaging data (ADNI dataset^21^). We demonstrated the goodness of our approach by running several tests based on 2D and 3D images, respectively, combined with tabular data and by comparing the results obtained by the multi-input model with the only-images and only-tabular models. Results show that enabling multi-input architectures in the FL framework improves the performance regarding both accuracy and F1 score concerning non-federated models while complying with data protection practices.

### Limitations and future work

The main goal of this work was to demonstrate the feasibility and effectiveness of horizontal federated multi-input ML models in the biomedical field. Hence, the centralized setting is not aggressively optimized, so the achieved model performance does not exceed the state of the art. However, the federated setting enables the virtual pooling of different and unshared data sources from multiple institutions, overcoming the “little data” issue while improving the generalization capability of the resulting model against any model trained using only data in a single institution.

The proposed approach does not consider the problem of missing views, which also affects clinical data processing. However, we are confident that the openness and flexibility of the proposed framework will foster research in the field, marking a step in data sharing and distributed processing.

Another limitation of the current study is the lack of an intermediate “validated federation” setting. This scenario would reuse the same 5-fold data split used to run the centralized experiments. Despite not being as realistic as the federated scenario presented here, it would add better comparable results between the centralized and federated settings and provide additional indicators to the current study.

Among the main future directions are the following.

• To propose a VFL setting where each client has a different type of input source and DL model. In particular, each participant in a federation can have various data types, such as images, tabular features, or text reports. In this scenario, models trained at different institutions from different data types should be somehow aggregated at the end of each round. Preliminary results show that aggregating only the identical architectural layers of different networks (particularly the classifier of two different convolutional neural networks [CNNs]) leads to a performance comparable to the typical case in which all model parameters are aggregated. Despite this new aggregation technique presenting the typical limitations of FL (i.e., layers must be identical to be aggregated), further investigation is required to analyze the weighting of the input features of the classifier.
• To explore the “validated federation” scenario described above for an additional perspective and to try to answer the question does aggregating the best-validated models lead to a better global model?
• To expand the hyper-parameter tuning phase, starting with parameters specifically related to training a federated model compared with a centralized one. In particular, we will test our proposed architecture with more than one epoch per round, enabling us to select the best model to simulate cross-validation and, ideally, achieve performance gain.

## Experimental procedures

### Resource availability

#### Lead contact

Requests for information and resources used in this article should be addressed to Walter Riviera (walter.riviera@univr.it).

#### Materials availability

This study did not generate new unique reagents.

### Implementation details

All the experiments for COVID-19 prognosis were performed at the HPC4AI^75^ facility of the University of Torino (node: 8 cores per CPU, AMD EPYC-IPBP, 1 NVIDIA A40 GPU). For FL experiments, we adopted OpenFL,^76^ the new framework for FL developed by the Intel Internet of Things Group (IOTG) and Intel Labs. FL experiments were executed on a distributed environment encompassing six collaborators (clients in the federation that train a global model on a local dataset) and one aggregator (aggregating the model updates received from collaborators), each running on the previously described node.

For the ADNI case study, all the experiments were performed on a 4-node cluster of dual-socket machines equipped with Intel Xeon Platinum 8380 CPU @ 2.30 GHz, with 40 physical cores per socket.

### Architecture

**Figure 3:**
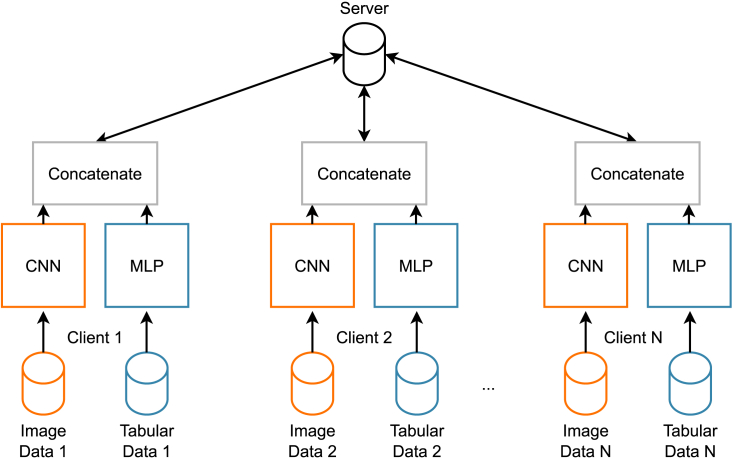
A general FL infrastructure with multi-input neural network models

Figure 3 displays the architecture of a multi-input NN. The general idea is to aggregate two different NNs trained on the same dataset using different data types. In particular, the ultimate goal is to aggregate a CNN^77^ and an MLP,^72^ respectively trained using as input features a set of images and a tabular data frame. CNN and MLP are used as feature extractors. We used ResNet-18^71^ as a reference model for the CNN and defined a custom MLP consisting of 3 hidden layers.

The architectures of both models are available on the project repository.^74^

Evaluation metric: for each experiment, we returned the test accuracy value defined as the ratio between correct guesses among all guesses; more precisely:

(Equation 1) $$\text{accuracy}=\frac{TP+TN}{TP+TN+FP+FN}$$

where *T* and *F* stand for true and false and *P* or *N* refers to positive or negative.

However, for biomedical applications, accuracy might not be enough to understand the goodness of a classifier. For a more in-depth analysis, we also calculated the F1 score for each experiment, which takes into account data unbalance. This metric is defined as the harmonic mean of precision and recall. Specifically, the metrics are defined as follows:

(Equation 2) $$\text{precision}=\frac{TP}{TP+FP}$$

(Equation 3) $$\text{recall}=\frac{TP}{TP+FN}$$

(Equation 4) $$F1-\text{score}=2*\frac{\text{precision}\;*\;\text{recall}}{\text{precision}+\text{recall}}$$

Model: models were trained by minimizing the binary cross-entropy loss with mini-batch gradient descent using the Adam optimizer with learning rate 1e−4 and OneCycleLR as scheduler. The local batch size was 8. The number of training epochs and FL rounds on the COVID-19 classification task was set to 100, while the Alzheimer’s detection task was set to 200.

### Datasets

We tested the multi-input NN on two tasks.

(1)Prognosis of COVID-19 disease from CXR, using the CoViD-CXR dataset.^20^
(2)Detection of AD from neuroimaging data, using the ADNI dataset.^21^

The federated setting emulates a realistic medical non-i.i.d. scenario, where each $C_{i}$ is hosted on an independent computing node using its dataset, contrasting with standard procedures where non-i.i.d distributions are often simulated by splitting a single source dataset hosted in a single machine.

### COVID-19 dataset

This task relied on real-world data of CXR and clinical parameters, divided into training and testing sets. Data were collected from six hospitals in emergency conditions during the first outbreak in Northern Italy in collaboration with Centro Diagnostico Italiano and Bracco Imaging. Due to the different data collection procedures, the distribution of image features varies across hospitals, leading to the well-known problem of non-i.i.d.ness.^30^^,^^78^ The CoViD-19 CXR dataset consists of 1,589 patients. Each of them is provided with a CXR and some clinical parameters (namely age, sex, positivity at admission, temperature, days of fever, cough, difficulty in breathing, white blood cell [WBC], red blood cell [RBC], C-reactive protein [CRP], glucose, lactate dehydrogenase [LDH], INR, PaO2, PaCO2, pH, high blood pressure, diabetes, dementia, BPCO, cancer, CKD, and respiratory failure). The dataset details are summarized in Table 8. Additional information about this dataset can be found at https://aiforcovid.radiomica.it/.

**Table 8:** Statistics of the CoViD-19 CXR dataset with class-balance percentages

Hospital	Samples	Positives (%)	Negatives (%)
A	120	85 (70.83)	35 (29.17)
B	104	59 (56.73)	45 (43.27)
C	151	81 (53.64)	70 (46.36)
D	139	76 (54.68)	63 (45.32)
E	101	55 (54.46)	46 (45.54)
F	974	546(56.06)	428 (43.94)

This dataset exhibits a clear quantity skew distribution because, as shown in Table 8, more than 60% of the data are stored in hospital F. However, recent works^30^^,^^78^ in FL literature show that quantity skew does not degrade the model’s performance because most FL algorithms, such as FedAvg,^17^ adopt a weighted averaging of the parameters. As a result, the distribution of samples (except for the quantity) is uniform among parties, which is the easiest setting. All the images, provided in JPEG format, were rescaled to 256 × 256. As for data augmentation, we performed random horizontal flips and random crops with a probability of 50%.

### ADNI dataset

Data used in the preparation of this article were obtained from the ADNI database (https://adni.loni.usc.edu). The ADNI dataset represents an ongoing, longitudinal, and multicenter study, the main landmark repository currently available for AD. Beginning in October 2004, ADNI has the primary goal of defining outcome measures to be used in clinical trials for assessing the treatment effectiveness in patients with AD. However, its scope has been further widened over the years, pointing to identifying early-diagnosis biomarkers in the pre-dementia stage. A comprehensive set of clinical, neuropsychological, neuroimaging (MRI and positron emission tomography), genetic, and biochemical data are currently collected in large cohorts of healthy elderly subjects, patients with mild cognitive impairment (MCI), and patients with AD. In particular, this study has been organized into different subsequent phases, the main ones being ADNI1 (2004–2011), ADNI2 (2011–2016), and ADNI3 (2016–ongoing), each of which has witnessed the enrollment of a significant number of new subjects over time and the progressive expansion of the adopted technologies and collected data.^21^^,^^79^ Up-to-date information is available at https://adni.loni.usc.edu.

We downloaded and used the 3D T1-weighted MRI scans acquired at baseline as imaging data for this study. We leveraged healthy control (CN) subjects and patients with AD for each ADNI set as two distinct classes. Details about the acquisition protocols regarding scanners, sequences, and corresponding parameters can be found at https://adni.loni.usc.edu/methods/documents/mri-protocols/. The coded information in the updated “ADNIMERGE.csv” file was retained to build the tabular features used to feed the models. More precisely, the following indicators were considered: age, gender, and APOE4 (ε4 allele of apolipoprotein E). The latter, in particular, represents the most decisive known genetic risk factor for AD and assumes either 0, 1, or 2 according to the number of ε4 alleles of the APOE gene. Subjects with incomplete data were removed, leading to the final samples reported in Table 4.

#### ADNI pre-processing

The individual 3D T1-weighted volumes were minimally pre-processed, including reorientation, bias-field correction and non-linear registration to the MNI152-2 mm standard space with dimensions of 91 × 109 × 91 (*fsl_anat* tool^80^). Data used to feed the models have also been normalized using the “min-max” scaling formula reported below.

For each input image *X*,

(Equation 5) $${\text{scaled}}_{X}=\frac{X-\min\left(X\right)}{\max\left(X\right)-\min\left(X\right)}$$

## Data Availability

The CoViD-CXR^20^ and ADNI datasets^21^ are publicly available. The code used for experimental evaluation is publicly available.^74^

## References

[1] Al-Issa Y., Alqudah A.M. (2022). A lightweight hybrid deep learning system for cardiac valvular disease classification. Sci. Rep..

[2] Mansour R.F., Alfar N.M., Abdel-Khalek S., Abdelhaq M., Saeed R.A., Alsaqour R. (2022). Optimal deep learning based fusion model for biomedical image classification. Expet Syst..

[3] Lai X., Zhou J., Wessely A., Heppt M., Maier A., Berking C., Vera J., Zhang L. (2022). A disease network-based deep learning approach for characterizing melanoma. Int. J. Cancer.

[4] Song Y., Ren S., Lu Y., Fu X., Wong K.K.L. (2022). Deep learning-based automatic segmentation of images in cardiac radiography: A promising challenge. Comput. Methods Progr. Biomed..

[5] Weston A.D., Korfiatis P., Kline T.L., Philbrick K.A., Kostandy P., Sakinis T., Sugimoto M., Takahashi N., Erickson B.J. (2019). Automated abdominal segmentation of ct scans for body composition analysis using deep learning. Radiology.

[6] Bhatt P., Sahoo A.K., Chattopadhyay S., Bhatt C. (2022). *Artificial Intelligence in Industrial Applications* ( 161–174).

[7] Liu X., Shan W., Li T., Gao X., Kong F., You H., Kong D., Qiao S., Tang R. (2021). A review of deep-learning-based medical image segmentation methods. BMC Cancer.

[8] D’Ascenzo F., De Filippo O., Gallone G., Mittone G., Deriu M.A., Iannaccone M., Ariza-Solé A., Liebetrau C., Manzano-Fernández S., Quadri G. (2021). Machine learning-based prediction of adverse events following an acute coronary syndrome (PRAISE): a modelling study of pooled datasets. Lancet.

[9] Gawehn E., Hiss J.A., Schneider G. (2016). Deep learning in drug discovery. Mol. Inform..

[10] Zhang L., Tan J., Han D., Zhu H. (2017). From machine learning to deep learning: progress in machine intelligence for rational drug discovery. Drug Discov. Today.

[11] Frid-Adar M., Diamant I., Klang E., Amitai M., Goldberger J., Greenspan H. (2018). Gan-based synthetic medical image augmentation for increased CNN performance in liver lesion classification. Neurocomputing.

[12] Zhao A., Balakrishnan G., Durand F., Guttag J.V., Dalca A.V. (2019). IEEE Conference on Computer Vision and Pattern Recognition, CVPR 2019.

[13] Sedik A., Iliyasu A.M., Abd El-Rahiem B., Abdel Samea M.E., Abdel-Raheem A., Hammad M., Peng J., Abd El-Samie F.E., Abd El-Latif A.A. (2020). Deploying machine and deep learning models for efficient data-augmented detection of covid-19 infections. Viruses.

[14] Cruciani F., Brusini L., Zucchelli M., Retuci Pinheiro G., Setti F., Boscolo Galazzo I., Deriche R., Rittner L., Calabrese M., Menegaz G. (2021). Interpretable deep learning as a means for decrypting disease signature in multiple sclerosis. J. Neural. Eng..

[15] Sello P., Bagula A.B., Ajayi O., Zitouni R., Agueh M., Houngue P., Soude H. (2019). *e-Infrastructure and e-Services for Developing Countries - 11th EAI International Conference, AFRICOMM 2019, Porto-Novo, Benin, December 3-4, 2019, Proceedings* vol. 311 of *Lecture Notes of the Institute for Computer Sciences, Social Informatics and Telecommunications Engineering*.

[16] Rosenbloom S.T., Smith J.R.L., Bowen R., Burns J., Riplinger L., Payne T.H. (2019). Updating HIPAA for the electronic medical record era. J. Am. Med. Inf. Assoc..

[17] McMahan B., Moore E., Ramage D., Hampson S., y Arcas B.A., Singh A., Zhu X.J. (2017).

[18] Mothukuri V., Parizi R.M., Pouriyeh S., Huang Y., Dehghantanha A., Srivastava G. (2021). A survey on security and privacy of federated learning. Future Generat. Comput. Syst..

[19] Yang Q., Liu Y., Cheng Y., Kang Y., Chen T., Yu H. (2019).

[20] Soda P., D’Amico N.C., Tessadori J., Valbusa G., Guarrasi V., Bortolotto C., Akbar M.U., Sicilia R., Cordelli E., Fazzini D. (2021). Aiforcovid: predicting the clinical outcomes in patients with covid-19 applying ai to chest-x-rays. an italian multicentre study. Med. Image Anal..

[21] Weiner M.W., Veitch D.P., Aisen P.S., Beckett L.A., Cairns N.J., Green R.C., Harvey D., Jack C.R., Jagust W., Morris J.C. (2017). The alzheimer’s disease neuroimaging initiative 3: Continued innovation for clinical trial improvement. Alzheimers Dement..

[22] Fu X., Zhang B., Dong Y., Chen C., Li J. (2022). Federated graph machine learning: A survey of concepts, techniques, and applications. SIGKDD Explor.

[23] Joshi M., Pal A., Sankarasubbu M. (2022). Federated learning for healthcare domain - pipeline, applications and challenges. ACM Trans. Comput. Healthc..

[24] Mårtensson G., Ferreira D., Granberg T., Cavallin L., Oppedal K., Padovani A., Rektorová I., Bonanni L., Pardini M., Kramberger M.G. (2020). The reliability of a deep learning model in clinical out-of-distribution MRI data: A multicohort study. Med. Image Anal..

[25] Schmidt A.M., Desai A.D., Watkins L.E., Crowder H.A., Black M.S., Mazzoli V., Rubin E.B., Lu Q., MacKay J.W., Boutin R.D. (2023). Generalizability of deep learning segmentation algorithms for automated assessment of cartilage morphology and mri relaxometry. J. Magn. Reson. Imag..

[26] Liang X., Nguyen D., Jiang S.B. (2021). Generalizability issues with deep learning models in medicine and their potential solutions: illustrated with cone-beam computed tomography (CBCT) to computed tomography (CT) image conversion. Mach. Learn, Sci. Technol..

[27] Lai C., Zhou S., Trayanova N.A. (2021). Optimal ecg-lead selection increases generalizability of deep learning on ecg abnormality classification. Philos. Trans. A Math. Phys. Eng. Sci..

[28] Nguyen D., Kay F., Tan J., Yan Y., Ng Y.S., Iyengar P., Peshock R., Jiang S. (2021). Deep learning-based COVID-19 pneumonia classification using chest CT images: Model generalizability. Front. Artif. Intell..

[29] Cruciani F., Altmann A., Lorenzi M., Menegaz G., Galazzo I.B. (2022). IEEE-EMBS International Conference on Biomedical and Health Informatics, BHI.

[30] Casella B., Esposito R., Cavazzoni C., Aldinucci M. (2022). Proc. of th 1st Italian Conference on Big Data and Data Science.

[31] Venkateswaran P., Isahagian V., Muthusamy V., Venkatasubramanian N. (2022). Fedgen: Generalizable federated learning. CoRR abs/2211. arXiv.

[32] Sarma K.V., Harmon S., Sanford T., Roth H.R., Xu Z., Tetreault J., Xu D., Flores M.G., Raman A.G., Kulkarni R. (2021). Federated learning improves site performance in multicenter deep learning without data sharing. J. Am. Med. Inf. Assoc..

[33] Silva R.,S.S., Gutman B.A., Romero E., Thompson P.M., Altmann A., Lorenzi M. (2019). 16th IEEE International Symposium on Biomedical Imaging, ISBI.

[34] Sheller M.J., Edwards B., Reina G.A., Martin J., Pati S., Kotrotsou A., Milchenko M., Xu W., Marcus D., Colen R.R., Bakas S. (2020). Federated learning in medicine: facilitating multi-institutional collaborations without sharing patient data. Sci. Rep..

[35] Poria S., Cambria E., Bajpai R., Hussain A. (2017). A review of affective computing: From unimodal analysis to multimodal fusion. Inf. Fusion.

[36] Sleeman W.C., Kapoor R., Ghosh P. (2023). Multimodal classification: Current landscape, taxonomy and future directions. ACM Comput. Surv..

[37] Yan X., Hu S., Mao Y., Ye Y., Yu H. (2021). Deep multi-view learning methods: A review. Neurocomputing.

[38] Pan L., Ji B., Wang H., Wang L., Liu M., Chongcheawchamnan M., Peng S. (2022). MFDNN: multi-channel feature deep neural network algorithm to identify COVID19 chest x-ray images. Health Inf. Sci. Syst..

[39] Spairani E., Daniele B., Signorini M.G., Magenes G. (2022). A deep learning mixed-data type approach for the classification of fhr signals. Front. Bioeng. Biotechnol..

[40] Tran T., Le T., Pham D., Hoang V., Khong V., Tran Q., Nguyen T., Pham C. (2018). 24th International Conference on Pattern Recognition, ICPR.

[41] Sanabria R., Caglayan O., Palaskar S., Elliott D., Barrault L., Specia L., Metze F. (2018). How2: A large-scale dataset for multimodal language understanding. CoRR abs/1811. arXiv.

[42] Tripathi S., Beigi H.S.M. (2018). Multi-modal emotion recognition on IEMOCAP dataset using deep learning. arXiv.

[43] Menze B.H., Jakab A., Bauer S., Kalpathy-Cramer J., Farahani K., Kirby J., Burren Y., Porz N., Slotboom J., Wiest R. (2015). The multimodal brain tumor image segmentation benchmark (BRATS). IEEE Trans. Med. Imag..

[44] Bekker A.J., Greenspan H., Goldberger J. (2016). 13th IEEE International Symposium on Biomedical Imaging, ISBI.

[45] Liu S., Liu S., Cai W., Che H., Pujol S., Kikinis R., Feng D., Fulham M.J., ADNI (2015). Multimodal neuroimaging feature learning for multiclass diagnosis of alzheimer’s disease. IEEE Trans. Biomed. Eng..

[46] Cao G., Wang Y., Zhang M., Zhang J., Kang G., Xu X. (2022). IEEE International Conference on Acoustics, Speech and Signal Processing, ICASSP 2022, Virtual and Singapore.

[47] Messner E., Fediuk M., Swatek P., Scheidl S., Smolle-Jüttner F.M., Olschewski H., Pernkopf F. (2020). Multi-channel lung sound classification with convolutional recurrent neural networks. Comput. Biol. Med..

[48] Tong T., Gray K., Gao Q., Chen L., Rueckert D. (2017). Multi-modal classification of alzheimer’s disease using nonlinear graph fusion. Pattern Recogn..

[49] Li W., Newitt D.C., Gibbs J., Wilmes L.J., Jones E.F., Arasu V.A., Strand F., Onishi N., Nguyen A.A.T., Kornak J. (2020). Bidirectional LSTM with self-attention mechanism and multi-channel features for sentiment classification. Neurocomputing.

[50] Chen P., Zou B., Belkacem A.N., Lyu X., Zhao X., Yi W., Huang Z., Liang J., Chen C. (2022). An improved multi-input deep convolutional neural network for automatic emotion recognition. Front. Neurosci..

[51] Sportisse A., Schmutz H., Humbert O., Bouveyron C., Mattei P.-A. (2023).

[52] Dolci G., Rahaman M.A., Chen J., Duan K., Fu Z., Abrol A., Menegaz G., Calhoun V.D. (2022). 22nd IEEE International Conference on Bioinformatics and Bioengineering, BIBE.

[53] Kang H., Xia L., Yan F., Wan Z., Shi F., Yuan H., Jiang H., Wu D., Sui H., Zhang C., Shen D. (2020). Diagnosis of coronavirus disease 2019 (COVID-19) with structured latent multi-view representation learning. IEEE Trans. Med. Imag..

[54] Huang S., Shi W., Xu Z., Tsang I.W., Lv J. (2022). Efficient federated multi-view learning. Pattern Recogn..

[55] Kang Y., Liu Y., Liang X. (2022). Fedcvt: Semi-supervised vertical federated learning with cross-view training. ACM Trans. Intell. Syst. Technol..

[56] Yang Y., Ye X., Sakurai T. (2022). ICMLC 2022: 14th International Conference on Machine Learning and Computing.

[57] Qayyum A., Ahmad K., Ahsan M.A., Al-Fuqaha A., Qadir J. (2022). Collaborative federated learning for healthcare: Multi-modal COVID-19 diagnosis at the edge. IEEE Open J. Comput. Soc..

[58] Che S., Kong Z., Peng H., Sun L., Leow A., Chen Y., He L. (2022). Federated multi-view learning for private medical data integration and analysis. ACM Trans. Intell. Syst. Technol..

[59] Al-Jarrah O.Y., Yoo P.D., Muhaidat S., Karagiannidis G.K., Taha K. (2015). Efficient machine learning for big data: A review. Big Data Res.

[60] Alam M.U., Rahmani R. (2023). Fedsepsis: A federated multi-modal deep learning-based internet of medical things application for early detection of sepsis from electronic health records using raspberry pi and jetson nano devices. Sensors.

[61] Zhao Y., Barnaghi P.M., Haddadi H. (2022). Seventh IEEE/ACM International Conference on Internet-of-Things Design and Implementation, IoTDI 2022.

[62] Bernecker T., Peters A., Schlett C.L., Bamberg F., Theis F.J., Rueckert D., Weiß J., Albarqouni S. (2022). Fednorm: Modality-based normalization in federated learning for multi-modal liver segmentation. arXiv.

[63] Aggarwal P., Mishra N.K., Fatimah B., Singh P., Gupta A., Joshi S.D. (2022). COVID-19 image classification using deep learning: Advances, challenges and opportunities. Comput. Biol. Med..

[64] Ozkaya U., Öztürk S., Barstugan M. (2020). Coronavirus (COVID-19) classification using deep features fusion and ranking technique. arXiv.

[65] Barstugan M., Ozkaya U., Öztürk S., Xhina E., Hoxha K. (2021). Proceedings of the 4th International Conference on Recent Trends and Applications in Computer Science and Information Technology.

[66] Sadat S.U., Shomee H.H., Awwal A., Amin S.N., Reza M.T., Parvez M.Z. (2021). IEEE International Conference on Systems, Man, and Cybernetics.

[67] Zhang Y.M., Wang S., Xia K., Jiang Y., Qian P. (2021). Alzheimer’s disease multiclass diagnosis via multimodal neuroimaging embedding feature selection and fusion. Inf. Fusion.

[68] Zhang D., Wang Y., Zhou L., Yuan H., Shen D., Alzheimer's Disease Neuroimaging Initiative (2011). Multimodal classification of alzheimer’s disease and mild cognitive impairment. Neuroimage.

[69] Gao X., Shi F., Shen D., Liu M. (2022). Task-induced pyramid and attention GAN for multimodal brain image imputation and classification in alzheimer’s disease. IEEE J. Biomed. Health Inform..

[70] Wang Y., Gu X., Hou W., Zhao M., Sun L., Guo C. (2023). Dual semi-supervised learning for classification of alzheimer’s disease and mild cognitive impairment based on neuropsychological data. Brain Sci..

[71] He K., Zhang X., Ren S., Sun J. (2016). IEEE Conference on Computer Vision and Pattern Recognition, CVPR 2016, Las Vegas.

[72] Zhang W., Yin Z., Sheng Z., Li Y., Zhang A., Rangwala H. (2022). KDD ’22: The 28th ACM SIGKDD Conference on Knowledge Discovery and Data Mining.

[73] Casella B., Esposito R., Sciarappa A., Cavazzoni C., Aldinucci M. (2023). Experimenting with normalization layers in federated learning on non-iid scenarios. arXiv.

[74] Casella B., Riviera W. (2023).

[75] Aldinucci M., Rabellino S., Pironti M., Spiga F., Viviani P., Drocco M., Guerzoni M., Boella G., Mellia M., Margara P. (2018). ACM Computing Frontiers.

[76] Reina G.A., Gruzdev A., Foley P., Perepelkina O., Sharma M., Davidyuk I. (2021).

[77] O’Shea K., Nash R. (2015). An introduction to convolutional neural networks. arXiv.

[78] Li Q., Diao Y., Chen Q., He B. (2022). 38th IEEE International Conference on Data Engineering, ICDE 2022.

[79] Gamal A., Elattar M.A., Selim S., Fournier-Viger P., Yousef A.H., Bellatreche L., Awad A., Wakrime A.A., Ouhammou Y., Aït-Sadoune I. (2022). *Advances in Model and Data Engineering in the Digitalization Era - MEDI 2022 Short Papers and DETECT 2022 Workshop Papers, Cairo, Egypt, November 21-24, 2022, Proceedings* vol. 1751 of *Communications in Computer and Information Science*.

[80] Jenkinson M., Beckmann C.F., Behrens T.E.J., Woolrich M.W., Smith S.M. (2012). Neuroimage.

